# Maternal dietary fibre intake results in sex-specific single-cell molecular changes in the heart of the offspring

**DOI:** 10.1042/CS20257187

**Published:** 2025-11-25

**Authors:** Chaoran Yang, Hamdi A. Jama, Malathi S.I. Dona, Gabriella E. Farrugia, Crisdion Krstevski, Charles D. Cohen, Alexander R. Pinto, Francine Z. Marques

**Affiliations:** 1 Hypertension Research Laboratory, Department of Pharmacology, Biomedical Discovery Institute, Faculty of Medicine, Nursing and Health Sciences, Monash University, Clayton, Australia; 2 Victorian Heart Institute, Monash University, Clayton, Australia; 3 Cardiac Cellular Systems Laboratory, Baker Heart and Diabetes Institute, Melbourne, VIC, Australia; 4 Centre for Cardiovascular Biology and Disease Research, La Trobe University, Melbourne, Victoria, Australia; 5 Baker Heart and Diabetes Institute, Melbourne, Australia

**Keywords:** cardiovascular disease, DOHaD, fibre, flow cytometry, microbiota, single-cell transcriptomics

## Abstract

Some types of dietary fibre undergo fermentation by the gut microbiome, producing microbial metabolites called short-chain fatty acids (SCFAs) – these are protective against cardiovascular disease (CVD). Emerging evidence suggests that maternal fibre intake also protects the offspring. Here, we aimed to determine whether delivery of SCFAs during pregnancy results in sex- and cell-specific molecular changes to the offspring’s heart. Female mice were subjected to high or low-fibre diets during pregnancy and lactation, while all offspring received a standard-fibre diet. We then studied the single-cell transcriptome (scRNA-seq, *n* = 16) and immune composition (fluorescence-activated cell sorting, *n* = 27) of the hearts and gut microbiome profiles (16S rRNA, *n* = 28) of six-week-old male and female offspring. Maternal fibre intake induced significant changes in the cardiac cellular and immunological landscapes, revealing sex-specific signatures at the single-cell level. High-fibre intake reduced the number of monocytes in the hearts of male offspring and the number of B cells in both female and male offspring. Cardiac fibroblasts in both male and female offspring of high-fibre intake dams showed an anti-fibrotic transcriptome. In contrast, only male offspring showed an anti-inflammatory transcriptome in macrophages and endothelial cells. Our findings suggest that high-fibre intake during pregnancy may induce a CVD-protective transcriptome (i.e., anti-fibrotic and anti-inflammatory), especially in male offspring. These findings underscore the relevance of maternal dietary choices during pregnancy influencing cardiovascular health outcomes in the offspring.

## Introduction

According to the Global Burden of Disease study, diet is a significant risk factor for overall disease morbidity and mortality, including the development of cardiovascular disease (CVD) [1,2]. While some nutrients, such as sodium, increase CVD risk, others, such as dietary fibre, reduce it [3]. Mechanisms of how sodium contributes to CVD are well-established [4]; nevertheless, we are still in the process of understanding the mechanisms involved in responses to fibre. However, a key concept is now clear: the benefits of dietary fibre happen via the gut microbiota [5]. Fibre is any carbohydrate that cannot be digested in the small intestine and has a minimum degree of polymerisation. Once they reach the large intestine, some types of fibre, such as resistant starches, are fermented by commensal bacteria [6]. This process releases short-chain fatty acids (SCFAs), metabolites almost exclusively produced by the gut microbiota, with known benefits to blood pressure and cardiac health [7–9].

Maternal nutrient intake and intrauterine exposures to nutrients and metabolites are fundamental determinants of postnatal outcomes [10]. Nutrient quality and accessibility during foetal life underpin the developmental origins of health and disease [10]. This hypothesis is supported by experimental, clinical and epidemiological studies, and results in the induction of cardio-metabolic and other non-communicable diseases [11]. Maternal nutritional constraint is a fundamental non-genetic factor regulating foetal development [11–13]. Nutritional and metabolite allocation during foetal development significantly impacts growth and, more importantly, epigenetically regulates offspring’s postnatal phenotype and physiological variation [12]. These can have profound long-term effects on offspring’s cardiovascular health [14,15]. Previous studies have demonstrated that maternal high-fibre intake can alter the gut microbiota in the offspring, leading to increased production of SCFAs, which improves cardiometabolic and immune health [16–20]. Evidence supports a role for epigenetic mechanisms (e.g., acetylation of natriuretic peptide genes, HDAC9 [18,19]) or direct signalling via G coupled-protein receptors (e.g., GPR41, GPR43 [16]).

There are significant sex differences in the development of CVD [21]. For example, females are usually protected against high blood pressure until they reach menopause, when they overtake the rate of hypertension relative to age-matched males [21]. Females may also have a lower blood pressure threshold for CVD than males, including for myocardial infarction and heart failure [22]. Thus, understanding whether maternal fibre intake can equally benefit female and male offspring is crucial for developing strategies that can prevent CVD in both sexes.

Single-cell RNA-sequencing (scRNA-seq) is a powerful technique that allows for the unbiased analyses of transcriptional changes at the single-cell level [23–25]. Over the past decade, the application of scRNA-seq has rapidly expanded, especially in CVD research [23–26]. Here, we aimed to understand the impact of maternal fibre intake on the offspring’s cardiac cellular and molecular landscape. We performed extensive flow cytometry and scRNA-seq on cardiac tissue from male and female offspring of dams fed either a high- or low-fibre diet, revealing several transcriptional changes in these offspring. We also explored the association between sex-specific cardiac transcriptional changes induced by maternal fibre intake and gut microbiome alterations in the offspring using 16S rRNA sequencing. Combined, these studies show the long-term impact of maternal fibre intake in preventing CVD, particularly in males.

## Methods

### Animal experiments

All animal experiments were approved by Monash Animal Ethics Committee (approval number 17465) in compliance with guidelines by the National Medical and Health Research Council of Australia. Male and female C57BL/6J mice were obtained from the Monash Animal Research Facility and housed in a specific pathogen-free (SPF) environment at Monash University. Once mice were mated and a plug was found, female mice were placed on either a high-resistant starch diet (referred to as the ‘high-fibre diet’, SF11-025) or a diet without resistant starch (referred to as the ‘low-fibre diet’, SF09-028), both of which were obtained from Specialty Feeds. All offspring were transitioned to a standard chow diet at weaning at three weeks of age. For scRNA-seq analysis, the offspring of 16 dams (*n* = 8 high-fibre, referred as ‘high-fibre offspring’, *n* = 8 low-fibre, ‘low-fibre offspring’) were studied.

### Flow cytometry

Hearts were immediately harvested from male and female offspring mice after euthanasia in a CO_2_ chamber at 6 weeks of age (total *n* = 27, 4–8/sex/diet). The hearts were then digested in 3 ml of 2 mg/ml collagenase IV (LS004188, Worthington Biochem) and 1 mg/ml dispase II (04942078001, Roche) at 37°C for 45 min, with trituration every 15 min. The resulting cell suspension was filtered through a 70 µm mesh. Cellular debris was removed by centrifugation at 200×g for 15 min at 4°C with no break. The supernatant was aspirated, and the cells were resuspended in 2% heat-inactivated F.C.S. and 0.9 mM CaCl2 in 1X HBSS. Cells were then stained with an antibody cocktail including: I-A/I-E (2G9, BD Biosciences), CD11b (M1/70, BD Biosciences), CD64 (a & b alloantigens, X54-5/7.1, BD Biosciences), CD146 (ME-9F1, BD Biosciences), CD31 (390, BD Biosciences), Ly6C (HK1.4, Biolegend), CD59a (REA287, Miltenyi Biotec), Ly6G (1A8, Biolegend), NK1.1 (PK136, Biolegend), CD39 (Duha59, Biolegend), CD90.2 (30-H12, BD Biosciences) and CD45 (30-F11, BD Biosciences). SYTOX™ Green Dead Cell Stain (S34860, Invitrogen) and eBioscience™ Calcein Blue AM Viability Dye (65–0855-39, Invitrogen) were used to identify live and metabolically active cells. Cells were acquired on the BD LSR Fortessa-X20 and analysed using FlowJo (version 10.5.3).

### Cardiac tissue extraction and scRNA-seq library preparation

Male and female offspring mice were euthanised in a CO_2_ chamber at six weeks of age (*n* = 8 for high-fibre offspring, *n* = 8 for low-fibre offspring). The detailed tissue extraction procedures were described in our previous report [27]. Briefly, hearts were rapidly harvested, and after enzyme digestion, the washed cell suspension was incubated with CD31 (390, BD Biosciences) and CD45 (30-F11, BD Biosciences) antibodies. The cells were then stained with Sytox™ Green (S34860, Invitrogen) and Vybrant™ DyeCycle™ (V10273, Invitrogen). Stained cells were sorted using a FACSAria™ III cell sorter (B.D.) to remove myocytes, defined as CD31+and CD45-. scRNA-seq libraries were prepared using the 10 x Genomics 3’ v2 chemistry kit and sequenced on the Illumina HiSeq platform. We prepared four libraries (total ~40,000 cells), divided between maternal fibre intake with a mix of male and female cells.

### Data processing of scRNA-seq

Cell-gene count matrices were generated from FASTQ files using CellRanger (version 7.2.0) with the mm10 release of the mouse genome, obtained from the 10X Genomics website in May 2024. Quality filtering was carried out following the methodology outlined by Sárvári et al. [28]. Initially, empty droplets were filtered out using the emptyDrops function of DropletUtils 1.24.0 [29]. Low-quality droplets, defined as those with more than 15% of reads mapping to mitochondrial DNA, fewer than 1000 unique molecular identifiers (UMIs), or fewer than 500 detected genes, were subsequently excluded from the analysis. Outliers identified through principal component analysis (PCA), calculated using Scater 1.32.0 [30], were also removed. Additionally, genes detected in fewer than 10 cells across at least 50% of the samples were discarded from the count matrix. Furthermore, genes encoding ambient RNA (ambient genes) were identified as those present in more than 0.1% of empty droplets with UMI counts between 1 and 10 and a total UMI count greater than 50. These ambient genes were excluded from the calculation of variable genes in Seurat.

### Biological sex identification

We then applied a method previously described [31] to determine the biological sex of each droplet. Briefly, droplets that expressed *Xist* but did not express any of the five Y chromosome genes (*Ddx3y, Eif2s3y, Gm29650, Kdm5d* and *Uty*) were classified as female. In contrast, droplets that did not express *Xist* but did express one or more Y chromosome genes were classified as male. Additionally, droplets with *Xist* expression below the median level observed in those identified as female but with Y chromosome gene expression above the 10th percentile of that seen in those identified as male were also classified as male. Droplets for which the biological sex could not be determined were excluded from further analysis.

### Downstream analysis of scRNA-seq

In the subsequent analyses, non-protein-coding genes were excluded. Droplets identified as doublets by DoubletFinder 2.0.4 [32] were also removed. Clustering was conducted using Harmony embeddings [33] within Seurat 4.4.0 [34], followed by annotation based on known biological markers. Trajectory (pseudo-time) analysis was performed using Monocle3 1.3.7 [35]. For the differential expressed genes (DEGs) analysis, genes present in fewer than 10% of droplets were excluded. Expression matrices were analysed using a zero-inflated model by MAST 1.28.0 [36]. Pathway overrepresentation analysis was performed using clusterProfiler [37]. For analyses of secretory proteins, a list of mouse ligand-receptor pairs obtained from a previous study was used to identify secretory proteins [23]. Co-expression network analysis was performed using hdWGCNA 0.4.3 [38].

### Faecal 16S rRNA-seq library preparation

The V4 region of bacterial 16S rRNA (*n* = 28, from studies above) was amplified by PCR to construct DNA libraries for 16S rRNA sequencing, as described previously [8,39]. For PCR amplification, 20 ng of faecal DNA was mixed with 515F and 926R primers and Platinum Hot Start PCR Master Mix in a Thermal Cycler (BioRad). Each library, with a total of 240 ng, was sequenced as 300 bp paired-end reads on the Illumina MiSeq platform.

### 16S rRNA-seq data processing

16S rRNA sequencing datasets were processed using QIIME2 2024.2.0 [40]. Quality control was performed with the denoise-single function, selecting 240 bp for forward reads and 200 bp for reverse reads. After evaluating the α-diversity saturation curve, one sample with a sequencing depth of less than 10,000 reads was excluded. Representative sequences were classified into taxonomic categories using a pretrained classifier (gg_2022_10_backbone.v4.nb.qza) from the QIIME2 website, with classification performed using the feature-classifier classify-sklearn function. A phylogenetic tree was constructed using the fragment-insertion sepp function and the ‘sepp-refs-gg-13–8.qza’ database from the QIIME2 repository. Differential abundance analysis was performed with MaAsLin2 version 1.18.0 [41] with a false discovery rate (FDR)-adjusted *P* value < 0.05 considered significant.

### Statistical analysis

Statistical analyses for flow cytometry and scRNA-seq data were performed using R version 4.4. Normally distributed data were analysed using two-way ANOVA test, or unpaired Welch *t*-tests when separately comparing two groups. Non-normally distributed data were analysed using the Wilcoxon–Mann–Whitney test. Cellular data are shown as mean ± standard error of mean (SEM). For high-dimensional scRNA-seq and 16S rRNA datasets, *P* values were adjusted using FDR by the Benjamini-Hochberg procedure. DEGs in scRNA-seq datasets were defined as log_2_FoldChange > 0.5 or < −0.5, with FDR < 0.05.

## Results

### Maternal fibre intake reduces the number of pro-inflammatory immune cells in the heart of the offspring

**Figure 1:**
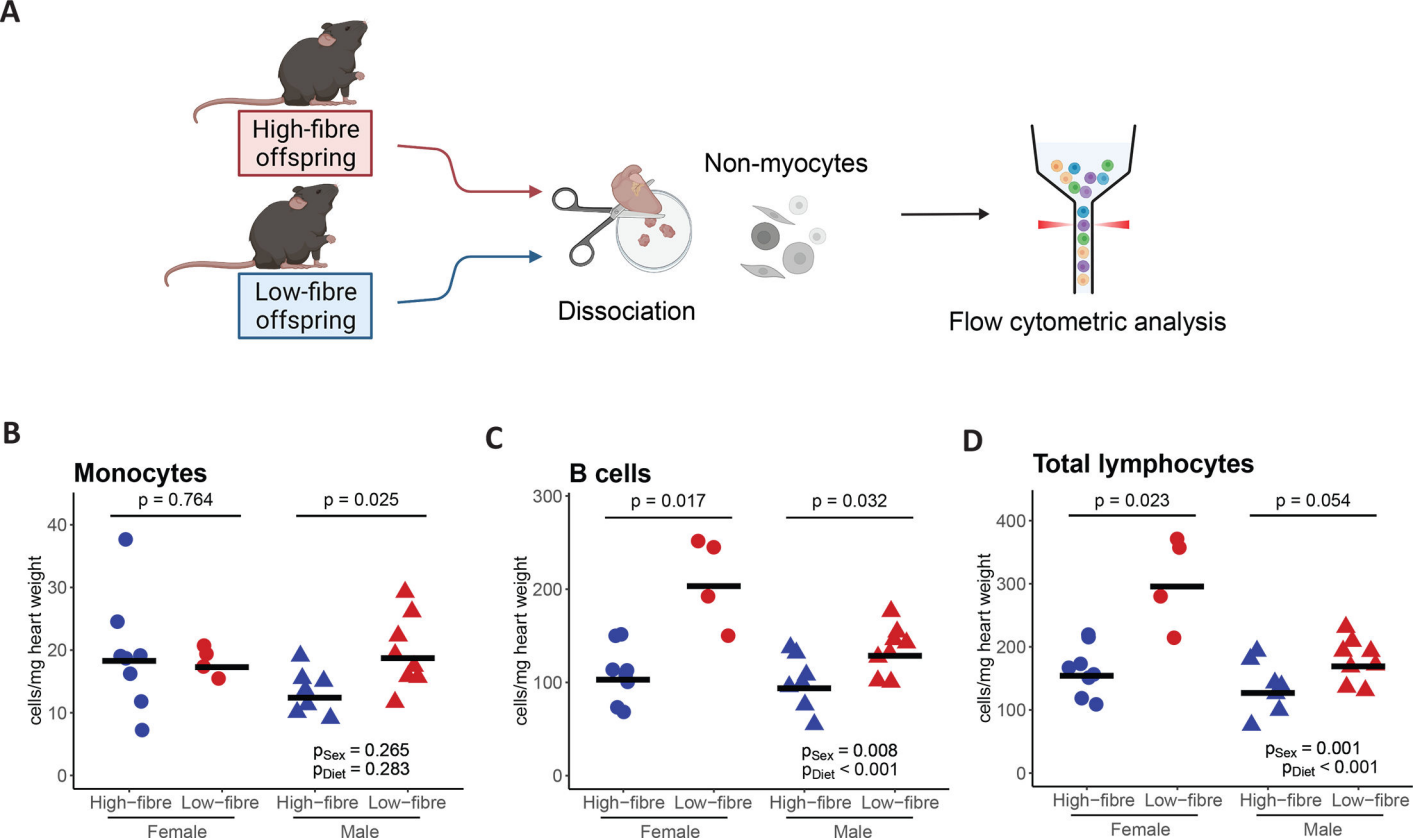
Overview of the cellular landscape changes in cardiac cells of high-fibre and low-fibre offspring. **A**. Schematic illustrating the experimental design for flow cytometric. **B-D**. heart weight-adjusted count of **B**. monocytes **C**. B cells and **D**. total lymphocytes in male and female high-fibre and low-fibre offspring. Normally distributed data were analysed using two-way ANOVA test or unpaired Welch *t*-tests when separately comparing two groups. Low-fibre male *n* = 8, low-fibre female *n* = 4, high-fibre male *n* = 7, high-fibre female *n* = 8.

To investigate the intergenerational effects of maternal fibre intake on the cellular composition of cardiac tissue, we applied high-dimensional flow cytometry to quantify and compare the numbers and ratios of 20 different non-myocyte cell types in male and female offspring from high-fibre and low-fibre diet-fed dams (Figure 1A, gating strategy in Supplementary Figure S1). Cardiac monocytes were reduced in male high-fibre offspring, while we observed no significant changes in female offspring (Figure 1B). We also observed a significant decrease in the heart weight-adjusted number of B cells, but no change in T cells, in both male and female high-fibre offspring compared with their low-fibre counterparts (Figure 1, Supplementary Figure S2). The total number of lymphocytes was significantly lower in female HF offspring, but not male offspring, compared with their low-fibre counterparts (Figure 1D). These findings suggest an anti-inflammatory effect of maternal fibre intake on cardiac tissue, particularly in male offspring. No significant differences were detected in the heart weight-adjusted numbers of other immune or non-immune cell types (Supplementary Figure S2, Supplementary Table S1). However, there were marked sex differences in the number of several cell types, including T cells, granulocytes, fibroblasts, residential mesenchymal cells, mural cells, smooth muscle cells (SMCs), Schwann cells, pericytes and Mcam+endothelial cells, which were overall lower in the male offspring, independent of diet (Supplementary Figure S2), which is in consistence with a previous study [42]. Low-fibre offspring had more B cells and lymphocytes and fewer Schwann cells than the high-fibre offspring, independent of sex (Figure 1C–D, Supplementary Figure S2).

### scRNA-seq identified eight major cell clusters

**Figure 2:**
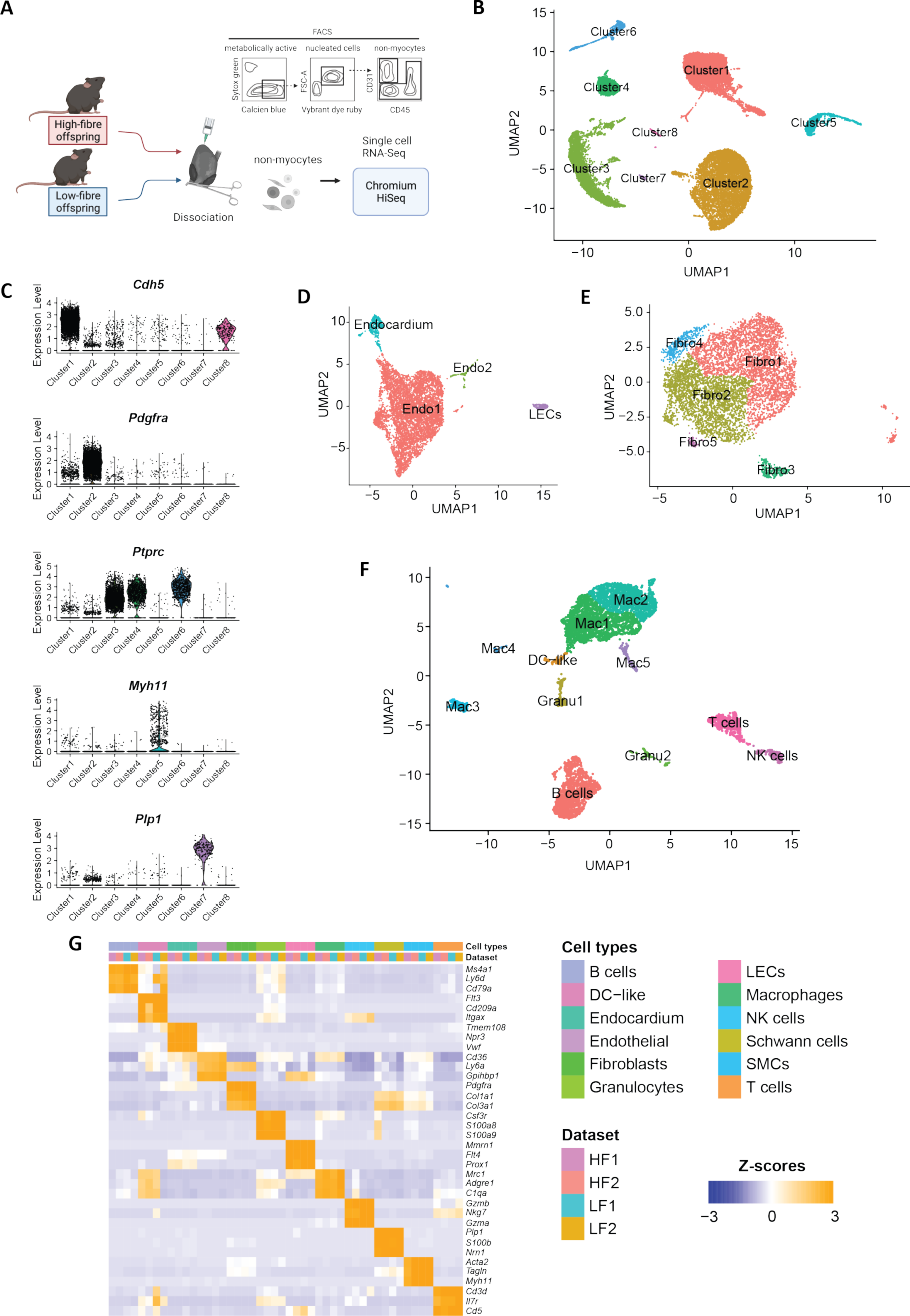
Overview of single-cell RNA-sequencing (scRNA-seq) datasets and the transcriptional landscape changes in cardiac cells of high-fibre and low-fibre offspring. **A**. Schematic illustrating the experimental design for scRNA-seq. **B**. Uniform manifold approximation and projection (UMAP) plot displaying eight primary cluster identified in the dataset. **C**. Distinctive marker genes expressed in primary clusters. **D–F**. UMAP plot displaying results of subclustering analyses in **D**. clusters 1 and 8 expression endothelial marker *Cdh5*. **E**. cluster 2 expressing fibroblast marker *pdgfra*. **F**. Clusters 3, 4 and 6 expressing immune marker *ptprc* (encoding CD45) **G**. heatmap showing markers specifically expressed in different cell types. HF, high-fibre offspring; LECs, lymphatic endothelial cells; LF, low-fibre offspring. *n* = 8 for high-fibre offspring, *n* = 8 for low-fibre offspring.

To investigate the intergenerational effects of maternal fibre intake on the transcriptional landscape of cardiac tissue, we performed scRNA-seq on the cardiac tissue of six-week-old offspring from dams fed a high-fibre and a low-fibre diet using the 10X Genomics Single Cell platform (Figure 2A) [18]. Following reference genome alignment and quality filtering (Supplementary Figure S3A-B), the biological sex of the sequenced cells was determined based on the expression of *Xist* and five Y chromosome genes (Supplementary Figure S3C-D). This resulted in 22,283 cells being selected for further analysis; 9,833 from male mice and 9,794 from female mice, while the biological sex of 2,656 cells could not be determined (Supplementary Table S2). Eight primary clusters were identified (Figure 2B). Among them, clusters 1 and 8 specifically expressed the endothelial marker *Cdh5* [43], while cluster 2 exhibited specific expression of the fibroblast marker *Pdgfra* [26]; clusters 3, 4 and 6 were characterised by the expression of the immune marker *Ptprc* (encoding CD45) [44]; cluster 5 specifically expressed smooth muscular cell (SMC) marker *Myh11* [45,46]*,* and cluster 7 specifically expressed Schwann cell marker *Plp1* marker (Figure 2C) [47]. We annotated cluster 5 as SMCs based on its specific expression of *Myh11*, *Tagln* and *Acta2* (Supplementary Figure S 4) [23,48]. Cluster 7 was annotated as Schwann cells due to its expression of *Nrn1*, *S100b* and *Plp1* (Supplementary Figure S 4) [23].

Subsequent analysis showed that *Cdh5*-expressing clusters 1 and 8 could be further subdivided into four clusters (Figure 2D, Supplementary Table S3). Two of these clusters specifically expressing endothelial markers *Cd36* and *Ly6a* [49,50] were named as Endo1 and Endo2 (Supplementary Figure 5A). Compared with Endo1, the Endo2 cluster highly expressed cell proliferating markers *Ube2c* and *Top2a [51]*. The remaining two clusters were annotated as endocardial cells, based on the expression of *Teme108* and *Npr3* [26] and lymphatic endothelial cells (LECs) characterised by *Prox1* and *Mmrn1* [52]*,* respectively (S upplementary Figure S5A)*.*

The *Pdgfra*-expressing cluster 2 could be divided into five clusters (Figure 2E, Supplementary Table S3). All these clusters highly expressed fibroblast markers *Pdgfra*, *Col1a1* and *Col3a1* (Supplementary Figure S4) [26,53] and were collectively designated as Fibro1-5. Fibro1 was characterised by high expression of *Pi16*, *Mfap5* and *Ly6a* (Supplementary Figure S5B), similar with a previously reported fibroblast population [54]. Fibro2 exhibited high expression of *Cxcl1*, *Hsd11b1* and *Cxcl14* (Supplementary Figure S5B), while Fibro3 was defined by high *Prg4*, *Wif1* and *Erbb4* (Supplementary Figure S5B). Fibro4 was distinguished by high expression of *Gpc6*, *Pcdh9* and *Arhgap24*, whereas Fibro5 showed elevated expression of *Nkain2*, *Inmt* and *Sntg1* (Supplementary Figure S5B).

*Ptprc* + clusters 3, 4 and 6 could be further divided into 11 clusters (Figure 2F, Supplementary Table S3). One cluster highly expressing B cell markers *Ms4a1* (encoding CD20), *Ly6d* and *Cd79a* [23] was annotated as B cells (Supplementary Figure S5 C). The cluster, characterised by high expression of* Gzma*, *Gzmb* and *Nkg7* [23], was identified as NK cells, while a cluster highly expressing *Cd5*, *Il7r* and *Cd3d* [23,24] was annotated as T cells. The subcluster expressing dendritic cell (DC) markers *Itgax*, *Cd209a* and *Flt3* (Supplementary Figure S5C) [55,56] was classified as DC-like cells. Two clusters expressing the granulocyte marker *Csf3r* were annotated as Gran1 and Gran2 (Supplementary Figure S4, S5C), [23]. When comparing Gran1 and Gran2, Gran1 highly expressed *Klra2* and *Itgal*, while Gran2 highly expressed *S100a8* and *S100a9* (Supplementary Figure S4, S5C) [23]. Five clusters expressing macrophage markers *C1qa*, *Adgre1* and *Mrc1* [23,55] (Supplementary Figure S4) were annotated as Mac1 to Mac5. Mac1 lacked *Lyve1* expression and highly expressed pro-inflammation markers *Cx3cr1* and *Cd74*, while Mac2 highly expressed *Lyve1* and tissue macrophage markers *Ccl6* and *F13a1* (S upplementary Figure S5C) [57]. Among the remaining macrophage clusters, Mac3 expressed *Sparc*, *Ly6c1* and *Fabp4*, Mac4 was characterised by expression of *Mgp*, *Gsn* and *Dcn*, and Mac5 expressed proliferation markers *Top2a* and *Ube2c* (S upplementary Figure S5C) [58]*.*

Subsequently, we aggregated clusters identified by cell types annotated and calculated the top 20 genes of every cell type (Figure 2G, Supplementary Tables S4-S5). Notably, the top-ranked marker genes identified in our analysis were consistent with previously reported findings [23].

### Maternal fibre intake results in sex-specific changes to the cardiac transcriptome

**Figure 3:**
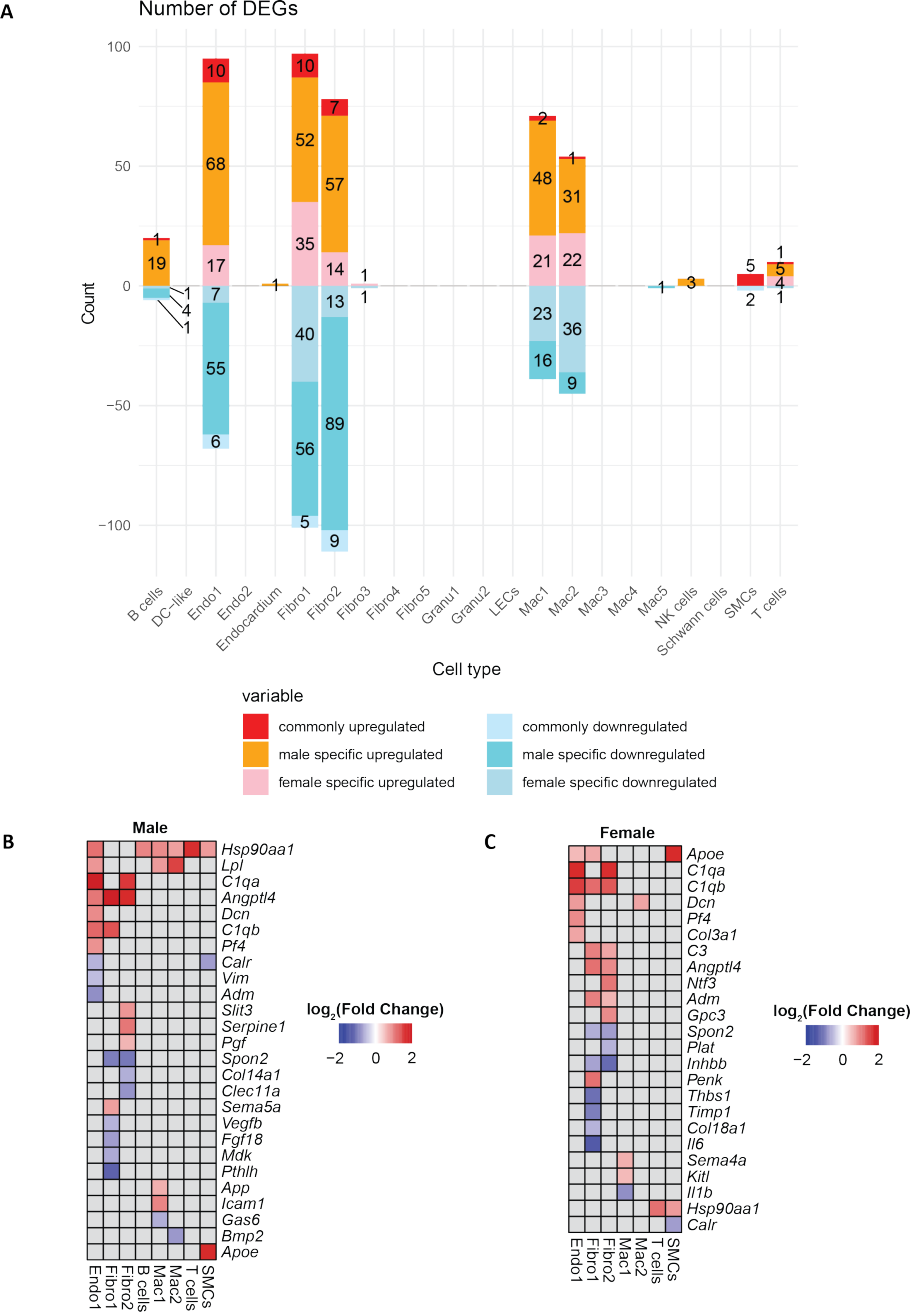
Transcriptional landscape changes in male and female high-fibre offspring. **A**. Bar plot comparing differentially expressed genes (DEGs) in male and female high-fibre offspring vs low-fibre offspring. Up-regulated genes are defined as genes with log2-transformed fold change > 0.5 and FDR < 0.05, and down-regulated genes are defined as genes with log2-transformed fold change < -0.5 and FDR < 0.05. **B**. Heatmap showing differentially expressed secretory protein-coding genes in male and female high-fibre offspring. HF, high-fibre offspring; LF, low-fibre offspring; Endo, endothelial; Fibro, fibroblasts; Gran, granulocytes; LECs, lymphatic endothelial cells; Mac, macrophages; SMCs, smooth muscle cells.

We then conducted sex-specific differential expression analysis in the identified clusters, comparing high-fibre with low-fibre offspring (Figure 3A, Supplementary Tables S6-S7). The majority of DEGs were observed in endothelial cells, fibroblasts and macrophage clusters. Furthermore, the number of DEGs in high-fibre offspring vs low-fibre offspring was higher in male offspring compared with female offspring, suggesting a stronger transcriptional response to maternal high-fibre intake in male offspring. Among these DEGs, some encode secretory proteins (Figure 3B–C). Specifically, the *Lpl* gene which encodes lipoprotein lipase, breaking down metabolic disorder-leading triglycerides, was exclusively up-regulated in endothelial and macrophage clusters in males [59]. Besides, anti-inflammatory *Slit3* [60] was only up-regulated in Fibro2 cluster in male high-fibre offspring.

**Figure 4:**
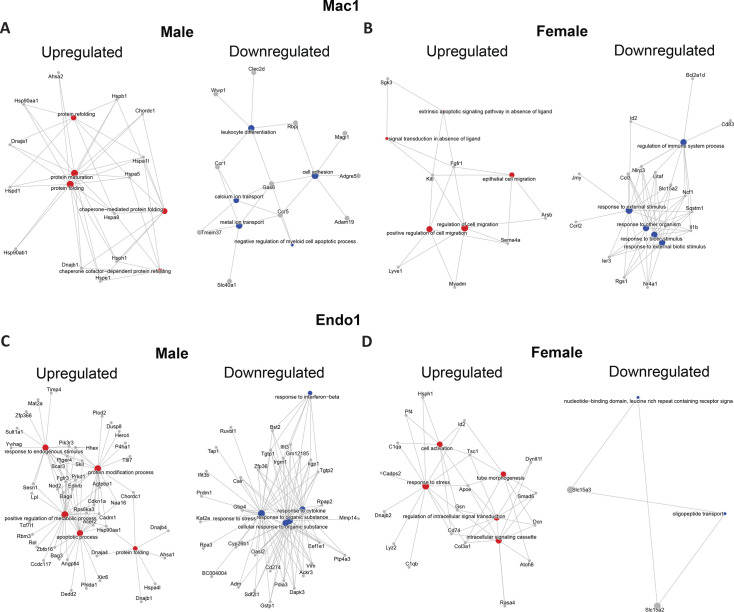
Pathway enrichment analysis of differentially expressed genes in male and female high-fibre offpsring. **A-B**. Mac1 and **C-D**. Endo1 population in male and female high-fibre offspring. Pathways enriched in up-regulated genes are in red, while pathways enriched in down-regulated genes are in blue.

Next, we performed pathway enrichment analysis for the DEGs identified (Supplementary Table S8). In the Mac1 cluster (Figure 4A), the term ‘leukocyte differentiation’ was only enriched in high rank in the down-regulated gene set of male high-fibre offspring (Supplementary Tables S8, S9). Notably, *Ccr1* and *Ccr5* genes, key for macrophage maturation [61], were only down-regulated in male high-fibre offspring. This observation may help explain the significantly lower cardiac monocyte count observed in male but not female high-fibre offspring (Figure 1B). Moreover, co-expression analysis identified a pro-inflammatory gene network (Supplementary Figure S6A). Consistent with the pathway enrichment results, this network was significantly down-regulated in the Mac1 and Mac2 clusters of male high-fibre offspring (Supplementary Figure S6B).

Interestingly, pathways related to protein folding, including *Dnajb1* [62] crucial for endoplasmic reticulum (ER) protein quality control, were enriched in male up-regulated genes in both Mac1 and Mac2 clusters of male high-fibre offspring (Figure 4A, Supplementary Figure S7A). In the female high-fibre offspring, different transcriptional changes were observed in macrophages. The term ‘response to external stimulus’ was enriched in high rank in down-regulated genes in the Mac1 cluster (Figure 3B), and the term ‘response to chemical stimulus’ was enriched in down-regulated genes in the Mac2 cluster (Supplementary Figure S7A). Moreover, pathways relevant to protein folding and quality control were also enriched in up-regulated genes in B cells of male high-fibre offspring (Supplementary Figure S7B).

A similar analysis in the endothelial population revealed that, in the Endo1 cluster, the terms ‘response to cytokine’ and ‘response to interferon-β’ were enriched in down-regulated genes of male high-fibre offspring, but not in females (Figure 4C–D, Supplementary Table S8). Besides, *Rel*, a key suppressor of the NF-κB pathway activation, was significantly up-regulated in male high-fibre offspring [63]. These findings suggest an anti-inflammation transcriptional landscape in the Endo1 cluster of male high-fibre offspring. Moreover, the term ‘positive regulation of metabolic process’ was enriched in high rank in up-regulated genes in male high-fibre offspring (Supplementary Table S8), including *Lpl* encoding triglyceride-breaking lipoprotein lipase. *Timp4*, which encodes tissue inhibitor of metalloproteinases 4 and regulates extracellular matrix remodelling [64], was also up-regulated. Moreover, genes coding proteins related to protein folding were found up-regulated in both male and female high-fibre offspring (Figure 4C–D).

**Figure 5:**
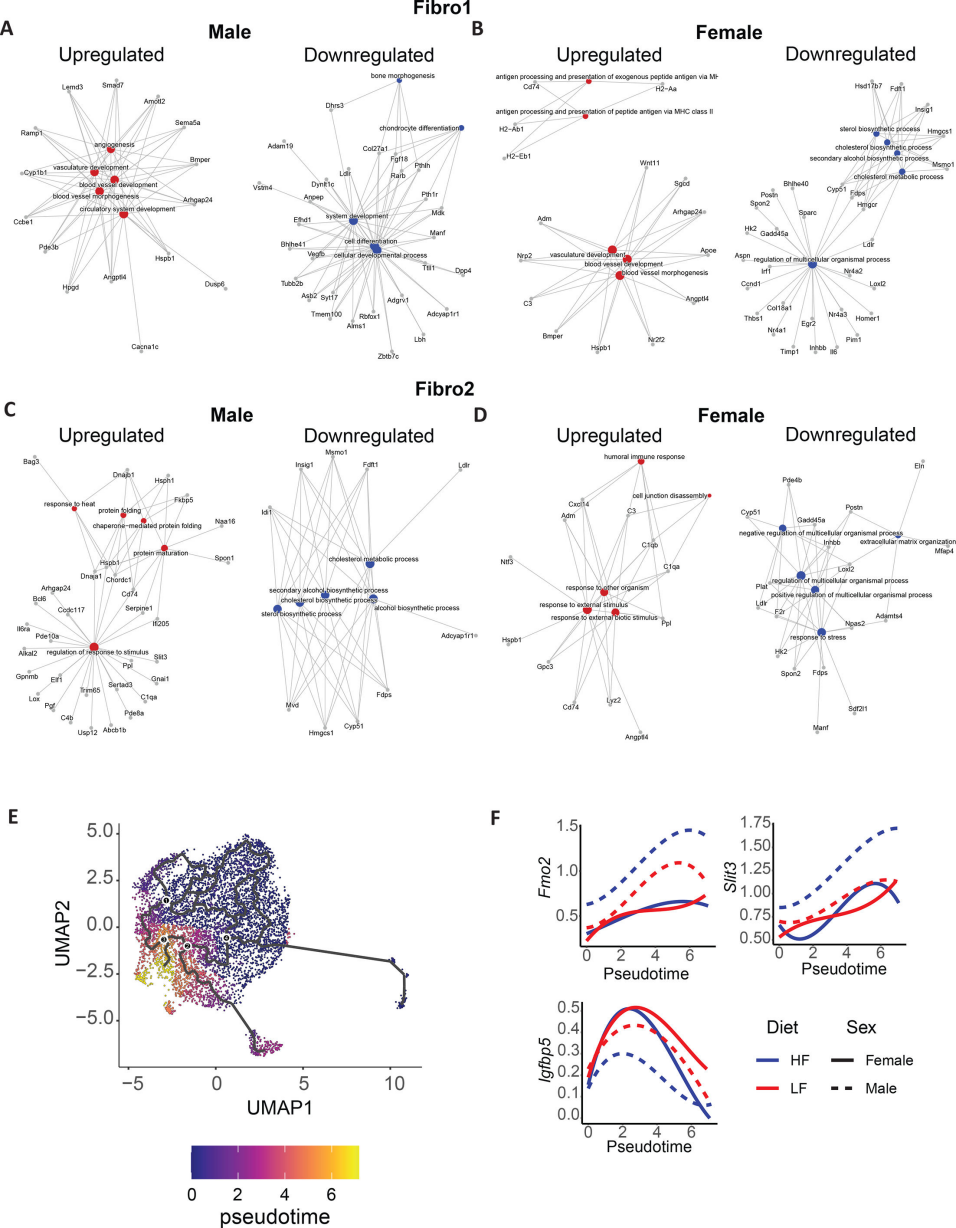
Transcriptional landscape changes in fibroblasts of male and female high-fibre offspring. A-B. Pathway enrichment analysis of differentially expressed genes. **A-B**. Fibro1 and **C-D**. Fibro2 population in male and female high-fibre offspring. Pathways enriched in up-regulated genes are in red, while pathways enriched in down-regulated genes are in blue. **E**. A plot showing the reconstructed differential trajectory by pseudo-time analysis. **F**. Expression profiles of *Fmo2*, *Slit3* and *Igfbp5* along the reconstructed differential trajectory. HF, high-fibre offspring; LF, low-fibre offspring.

Subsequent analysis in fibroblast populations demonstrated that, in Fibro1, the term ‘cell differentiation’, including *Ldlr and Manf,* was enriched in high rank in down-regulated genes in male high-fibre offspring (Figure 5A, Supplementary Tables S8, S9). *Ldlr* encodes the low-density lipoprotein receptor, while *Manf* is associated with pro-fibrotic and pro-inflammatory processes [65]. In contrast, the term ‘cholesterol metabolic process’, which also includes *Ldlr*, was only enriched in down-regulated genes in female high-fibre offspring (Figure 5B, Supplementary Table S8). Moreover, female and male high-fibre offspring had both up-regulation of ‘vasculature’ and ‘blood vessel development’ pathways in high rank (Figure 5A, Supplementary Tables S8, S9).

As for the Fibro2 cluster, terms related to protein folding were enriched in male high-fibre offspring up-regulated genes (Supplementary Table S8). Given that ER stress caused by protein misfolding is a key driver of fibrosis [66], this finding may provide a mechanistic explanation for the reduced fibrosis observed in high-fibre offspring following angiotensin II challenge [18,67]. Furthermore, terms related to cholesterol metabolism were enriched in male high-fibre offspring down-regulated genes (Figure 5C, Supplementary Table S8). Interestingly, by contrast, in the Fibro1 cluster, cholesterol metabolism pathways were only enriched in female (Supplementary Table S8). The term ‘extracellular matrix organisation’ was enriched in high rank (ranked fifth) in female high-fibre offspring down-regulated genes, suggesting an anti-fibrotic transcriptome (Figure 5D), consistent with our previous findings of reduced fibrosis in high-fibre offspring [18]. Conversely, the term ‘extracellular matrix organisation’ was ranked 31st in male high-fibre offspring down-regulated genes (Supplementary Table S9). Supporting this, a co-expression network associated with pro-fibrotic genes was significantly down-regulated in fibroblast clusters of both male and female high-fibre offspring (Supplementary Figure S6B-C), further reinforcing the results of pathway enrichment analysis in fibroblast populations.

Overall, these findings indicate transcriptional changes related to metabolism and fibrosis regulation in fibroblasts of both male and female high-fibre offspring. Although the specific clusters in which these genes were differentially expressed, as well as the ranks of their enrichment, differed between male and female offspring (Supplementary Table S9).

Because pathway enrichment analysis revealed that fibrotic pathways were consistently down-regulated in both male and female offspring (Supplementary Table S9), we next examined whether the reduction in pro-fibrotic gene expression was greater in one sex of offspring. To address this, we performed pseudo-time analysis to reconstruct the differentiation trajectory of fibroblast population. This analysis can help us understand the progression of fibrosis and pinpoint factors that may drive it over time. The results indicated that Fibro1 cluster gradually transforms into other cell types (Figure 4E, Supplementary Figure S9). On the reconstructed trajectory, the expression of progenitor markers *Pi16* and *Dpp4* decreased, while the expression of the mature fibroblast marker *Penk* increased (Supplementary Figure S9). Notably, the fibrosis-inhibiting *Fmo2* and anti-inflammatory *Slit3* expression were significantly up-regulated during fibroblast differentiation exclusively in male high-fibre offspring. By contrast, *Igfbp5*, which is a pro-fibrotic factor [68], was suppressed during fibroblast differentiation exclusively in male high-fibre offspring. These findings further support the fibrosis-suppressive effects of maternal fibre intake in male offspring (Figure 4F, Supplementary Table S9).

### Maternal fibre intake alters the gut microbiome of male offspring

The gut microbiota produces SCFAs through the fermentation of dietary fibre, a key mechanism through which dietary fibre contributes to cardiovascular health [8]. Moreover, our previous study demonstrated that maternal fibre intake modified the gut microbiome composition in the offspring [18]. What remained unclear was if maternal fibre intake would have differential effects on the gut microbiome of male versus female offspring, potentially contributing to observed sex-specific differences in cardiac transcriptional responses to maternal fibre intake, or whether the changes take place in the downstream mechanisms.

**Figure 6:**
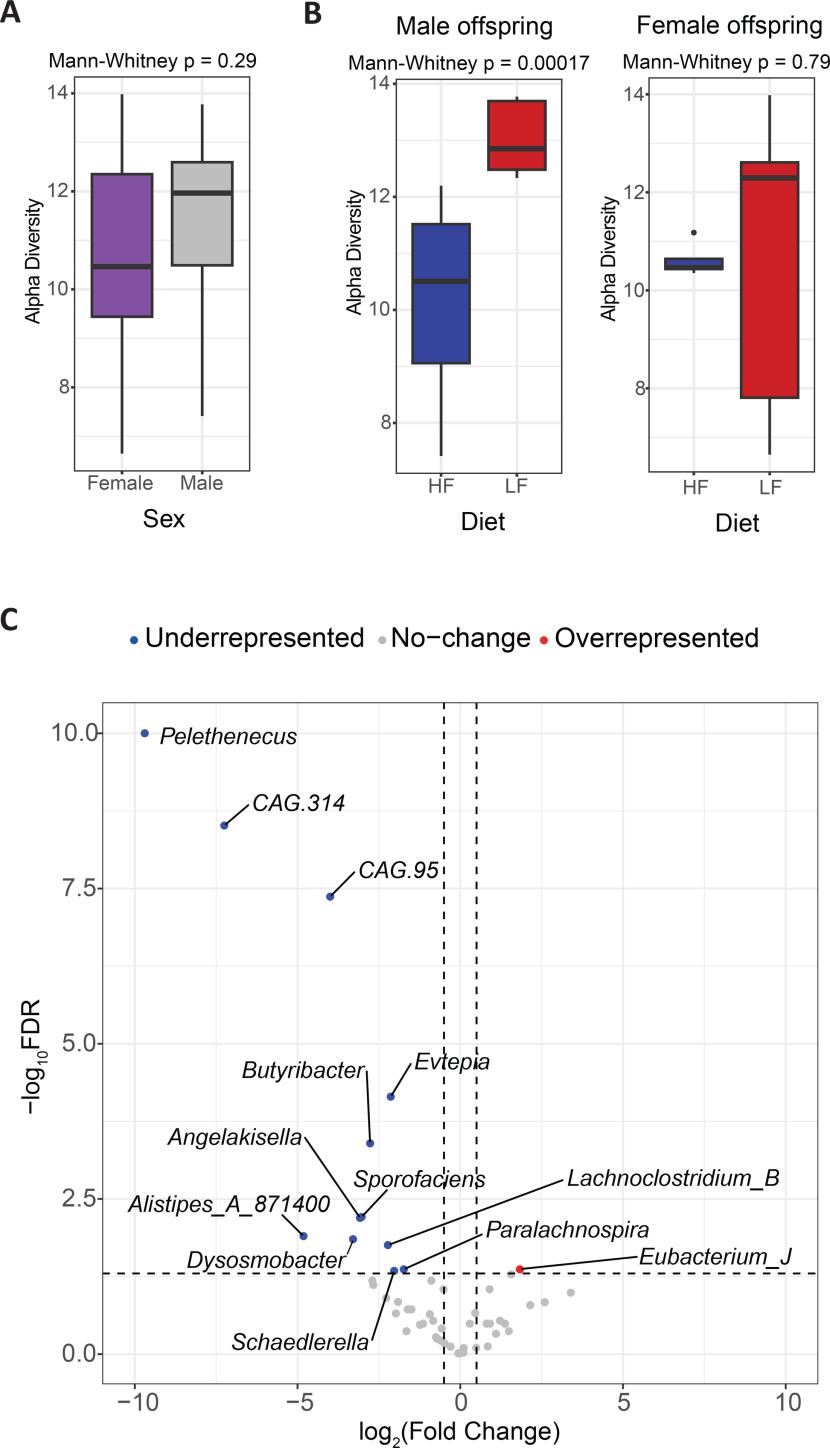
Gut microbiome changes in male and female high-fibre offspring. **A-B**. Alpha diversity (faith’s phylogenetic diversity) comparison of **A**. Male versus female offspring, and **B**. Male (left) and female (right) high-fibre (HF) and low-fibre (LF) offspring. **C**. Volcano plot showing overrepresented and underrepresented genera in male high-fibre offspring compared with low-fibre offspring. Sample size: *n* = 4 female high-fibre offspring, *n* = 9 male high-fibre offspring, *n* = 7 female low-fibre offspring, *n* = 7 male low-fibre offspring.

To test this hypothesis, we analysed the gut microbiota of high-fibre and low-fibre offspring of both sexes using 16S rRNA sequencing (Supplementary Figure S10). While sex as an independent factor did not significantly impact the α-diversity of the gut microbiome (Figure 6A), male high-fibre offspring exhibited significantly lower α-diversity than their low-fibre counterparts (Figure 6B). This is consistent with our previous findings that fibre-induced changes in gut pH can enhance the abundance of certain fibre-fermenting bacteria [69], which may lead to reduced α-diversity. Furthermore, while no significant differences were observed at the genus level between female high-fibre and low-fibre offspring, 12 genera were significantly underrepresented in male high-fibre offspring (Figure 6C), particularly the SCFA-producing genus *Eubacterium,* which was overrepresented (Figure 6C, Supplementary Table S10). These findings indicate that maternal fibre intake may impact the gut microbiome of male offspring.

## Discussion

There is a growing body of research focusing on sex differences in CVD; however, discrepancy still exists in our understanding of the biology of CVD between the sexes. In this study, we investigated the effects of maternal fibre intake on the cardiac cellular and transcriptional landscape using flow cytometric and scRNA-seq, focusing on sex differences in healthy animals. Our analysis revealed a male-specific anti-inflammatory transcriptome in macrophages and endothelial cells, and an anti-fibrosis transcriptome in both male and female offspring of high-fibre intake dams (Supplementary Tables S8-S9). However, the expression of some anti-fibrotic genes like *Fmo2* and *Slit3* was still stronger in the male high-fibre offspring. Moreover, changes in the gut microbiome were more prevalent in male offspring, which showed higher abundance of SCFA-producing bacteria induced by maternal fibre intake.

Chronic inflammation is a well-known biomarker of ageing [70,71] and is a significant risk factor for CVD in the elderly [72,73]. Our study showed a down-regulation of genes encoding pro-inflammatory chemokines, particularly *Ccr1 and Ccr5*, in the Mac1 cluster of male offspring from high-fibre diet-fed dams. Chronic inflammation is also associated with fibrosis, which adversely impacts CVD prognosis [74]. Our pseudo-time analysis of the fibroblast population reveals a progressive increase in the expression of anti-inflammatory factors during the dynamics of the fibroblast population. These findings suggest a role for these anti-inflammatory factors in mitigating fibrosis in this model. This is consistent with previous findings that maternal fibre intake resulted in lower fibrosis and inflammation in the offspring; however, only male mice were studied [18].

In our study, we observed notable transcriptional changes in endothelial cells beyond the down-regulation of atherosclerosis-inducing, pro-inflammatory genes such as *Ackr3* [75]. The reduced transcriptional activity of endothelial cells in male offspring of high-fibre intake dams within the context of dilated cardiomyopathy pathology further indicates potential cardiovascular benefits associated with high-fibre intake. Notably, the effects of some differentially expressed genes in endothelial cells present a complex picture. For example, while some studies have linked up-regulation of galectin-3, encoded by *Lgals3*, to cardiac dysfunction [76], other research suggests that galectin-3 could show anti-inflammatory and anti-fibrotic effects by recruiting macrophages [77,78]. Notably, *Lgals3* is down-regulated in endothelial cells under dilated cardiomyopathy pathology [79], which raises the possibility that the moderate up-regulation of *Lgals3* in endothelial cells could benefit cardiac health.

Sex-specific differences in the intergenerational effects of maternal diet have been recognised for some time. Some of the earliest studies on the intergenerational impact of maternal diet already reported that male and female offspring respond differently to maternal low-protein diets [80,81]. However, intergenerational sex-specific differences in the context of fibre, the gut microbiome and SCFAs remain elusive. While we did not observe sex differences in a randomised clinical trial where untreated hypertensive patients were treated with SCFAs [9], our study observed male-specific alterations in the gut microbiome due to maternal fibre intake, which aligns with findings from other studies reporting some sex differences in gut microbiota [82,83]. This could result in increased SCFA production and, thus, improved cardiovascular health, as observed in SCFA interventions [7,8]. A previous study showed that removing sex hormone differences by gonadectomy mitigated the sex-dependent differences in the non-myocyte cellular landscape of the heart [42]. This suggests that hormonal differences between males and females may contribute to some of the sex differences observed in our study. However, understanding their direct impact on these changes was outside the scope of this study. Moreover, the effect of prenatal epigenetic programming, such as DNA methylation, varies between sexes [84–86]. Male and female foetuses may respond differently to prenatal stress induced by maternal diet, indicating a distinct epigenetic landscape between male and female offspring of high-fibre intake mothers, which warrants further investigation.

We acknowledge this study has limitations. Firstly, current scRNA-seq techniques are still limited in accurately measuring transcriptional changes in minor cell populations such as Schwann cells, DCs and SMCs. Thus, these cells were not accurately represented in our scRNA-seq dataset. Due to the size of cardiomyocytes and, thus, the requirement of preparing a cardiomyocyte-specific library, these cells were not present in our library. Although they make up most of the heart volume, they represent only 25–35% of the total cell count [87]. Moreover, inflammation and fibrosis, both key traits of heart disease, are driven by non-myocytes such as fibroblasts and macrophages, which were represented in our dataset. Although we identified male-specific cardiac transcriptional changes associated with alterations in the gut microbiome, we did not establish a causal relationship between these gut microbiome changes and the observed transcriptional shifts. Future studies employing germ-free mouse models are necessary to clarify this causal link. Finally, as previously mentioned, the mechanisms underlying the observed sex differences remain unclear, and further investigation is needed.

In conclusion, maternal high-fibre intake alters the gut microbiome and reduces anti-inflammatory and anti-fibrotic transcriptional changes in the offspring’s cardiac tissue, particularly male offspring. These findings underscore the long-term effects of maternal diet, particularly for male offspring, and highlight the importance of increasing dietary fibre intake during pregnancy.

**Clinical perspectives**

- Emerging evidence suggests maternal fibre intake protects the offspring against cardiovascular disease. However, whether maternal fibre intake results in sex- and cell-specific molecular changes with cardiovascular benefits to the offspring is still unknown.
- High-fibre intake during pregnancy induces a cardiovascular-protective transcriptome, including the expression of cardiovascular protective secretory protein-coding genes, especially in the male offspring.
- These findings underscore the importance of maternal dietary choices in affecting offspring cardiovascular health outcomes.

## Supplementary material

online supplementary material 1

online supplementary material 2

## Data Availability

The 16S rRNA-seq data in this article are deposited to https://doi.org/10.5281/zenodo.14523136, and the scRNA-seq data are in GenBank Nucleotide Database with access number PRJNA1103151.

## Open Access

This article has been published open access under our Subscribe to Open programme, made possible through the support of our subscribing institutions, learn more here: https://portlandpress.com/pages/open_access_options_and_prices#conditional

## Abbreviations

CVD: cardiovascular disease
DEG: differentially expressed genes
SCFA: short-chain fatty acid
UMAP: Uniform Manifold Approximation and Projection
scRNA-seq: single cell RNA-seq
