# Supplementary material for: Maternal dietary fibre intake results in sex-specific single-cell molecular changes in the heart of the offspring
Source: Clin Sci (Lond). 2025 Nov 25;139(22):1527–42. doi: 10.1042/CS20257187 (PMC12751069; doi:10.1042/CS20257187)
Supplement: online supplementary material 1 [file CS-139-22-CS20257187-s001.docx]

**Online Supplementary Figures to**

**Maternal dietary fibre intake results in sex-specific single-cell molecular changes in the heart of the offspring**

**Running title:** Intergenerational sex-specific cardiac changes

Chaoran Yang^1,2^, Hamdi A. Jama^1^, Malathi S.I. Dona^3,4^, Gabriella E. Farrugia^3,4^, Crisdion Krstevski^3,4^, Charles. D. Cohen^3,4^, Alexander R. Pinto^3,4^, Francine Z. Marques^1,2,5^*

^1^Hypertension Research Laboratory, Department of Pharmacology, Biomedical Discovery Institute, Faculty of Medicine, Nursing and Health Sciences, Monash University, Clayton, Australia; ^2^Victorian Heart Institute, Monash University, Clayton, Australia; ^3^Cardiac Cellular Systems Laboratory, Baker Heart and Diabetes Institute, Melbourne, VIC, Australia; ^4^Centre for Cardiovascular Biology and Disease Research, La Trobe University, Melbourne, Victoria, Australia; ^5^Baker Heart and Diabetes Institute, Melbourne, Australia.

***Corresponding author**: Prof Francine Marques, Hypertension Research Laboratory, Victorian Heart Institute, Level 2, Victorian Heart Hospital, 631 Blackburn Road Clayton, VIC 3168 Monash University, Melbourne, Australia, Phone: +61-03-7511 1864. E-mail: [francine.marques@monash.edu](mailto:francine.marques@monash.edu)

**Online supplementary figures**


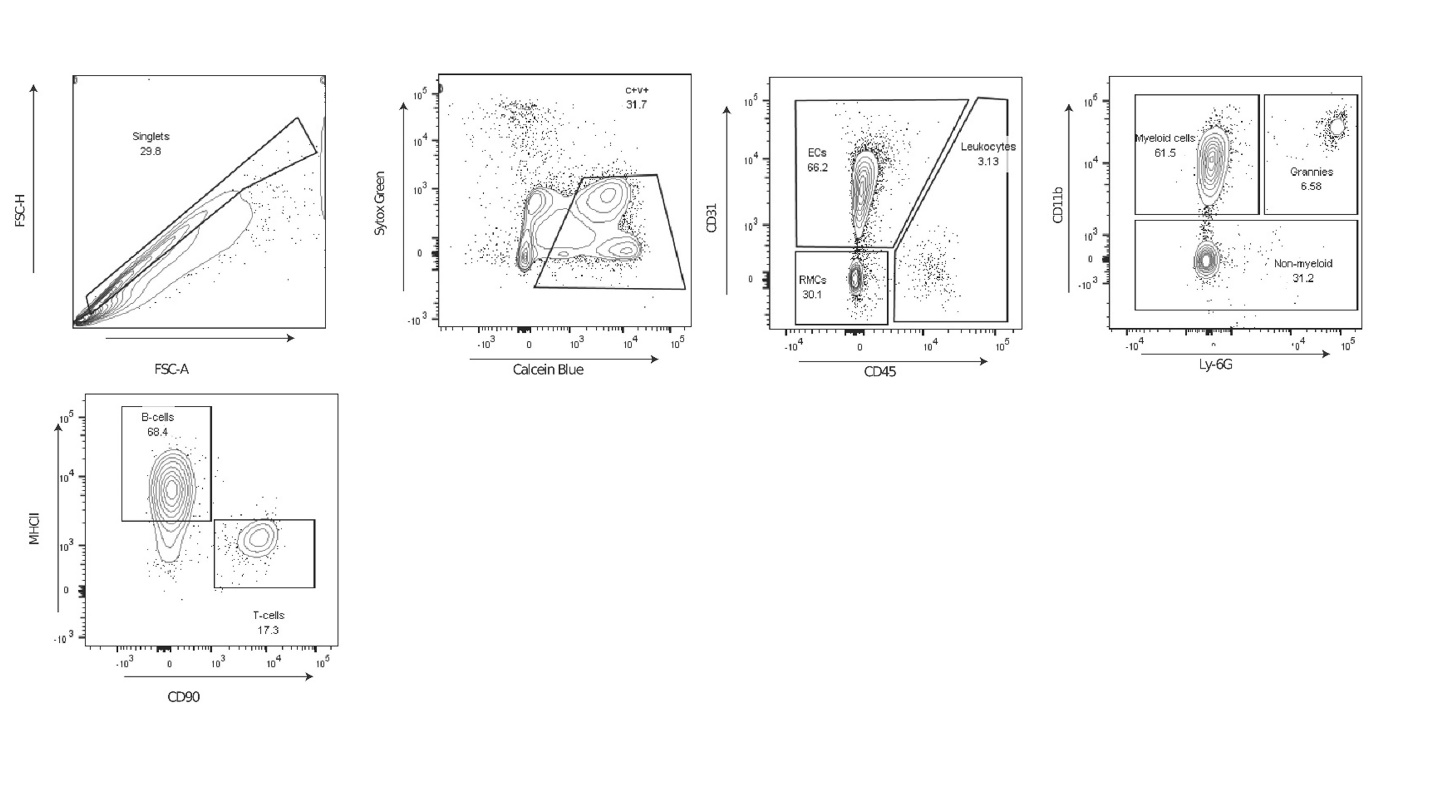


**Figure S1 | Gating strategy of flow cytometric.**


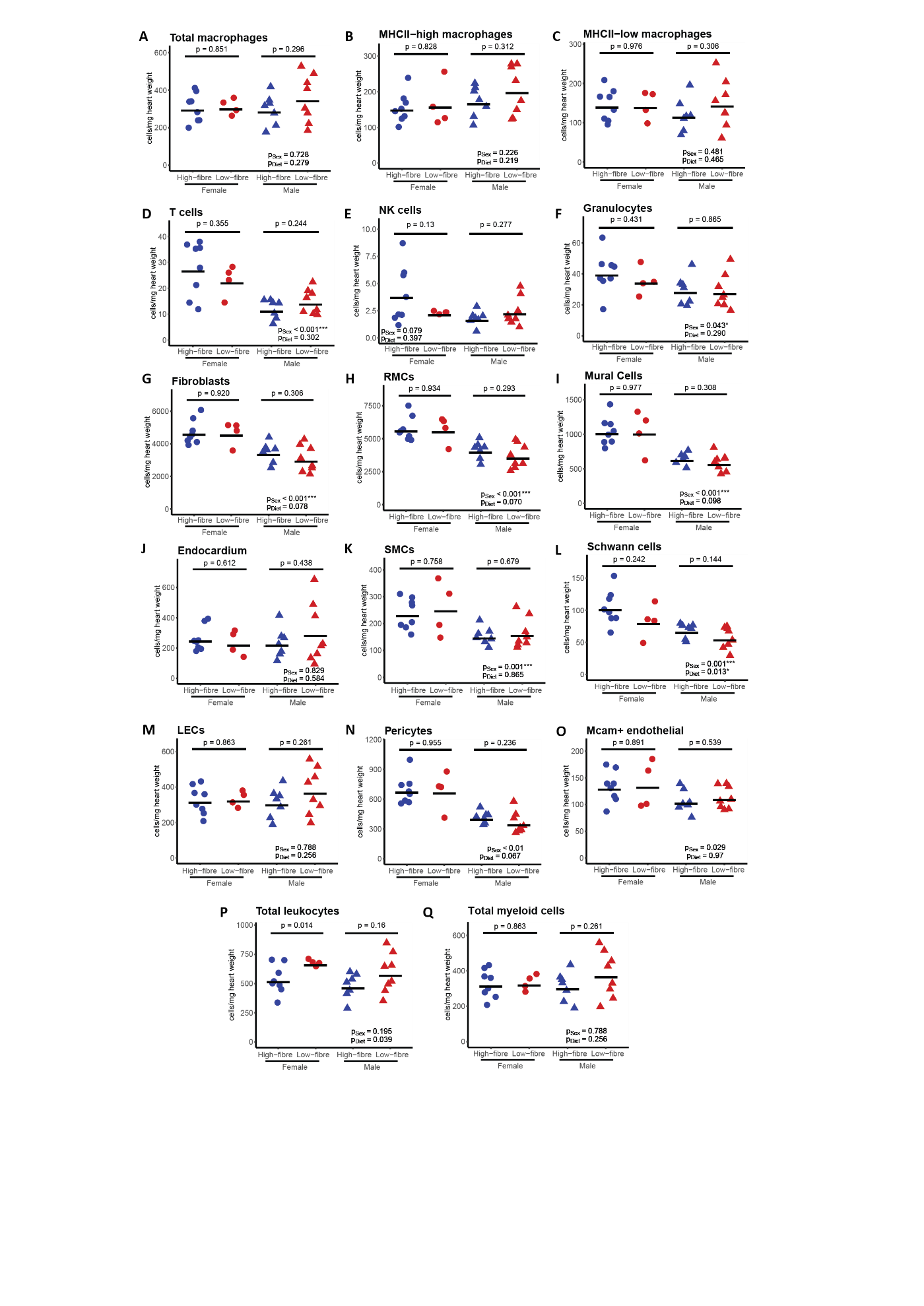


**Figure S2 | Fluorescence-Activated Cell Sorting (FACS) results showing heart weight-adjusted cell counts A.** Total macrophages. **B.** MHCII-high macrophages. **C.** MHCII-low macrophages **D.** T cells. **E.** Natural killer (NK) cells. **F.** Granulocytes. **G.** Fibroblasts. **H.** Residential mesenchymal cells (RMC). **I.** Mural cells. **J.** Endocardium. **K.** Smooth muscle cells (SMCs). **L.** Schwann cells. **M.** lymphatic endothelial cells (LECs) **N.** Pericytes. **O.** Mcam+ endothelial cells. **P.** Total leukocytes. **Q.** Total myeloid cells between male and female high- and low-fibre offspring. P_sex_ and P_diet_ were calculated by 2-way ANOVA test, while p-values comparing two subgroups were calculated by Welch t-test. Low fibre male n=8, low fibre female n=4, high fibre male n=7, high fibre female n=8.


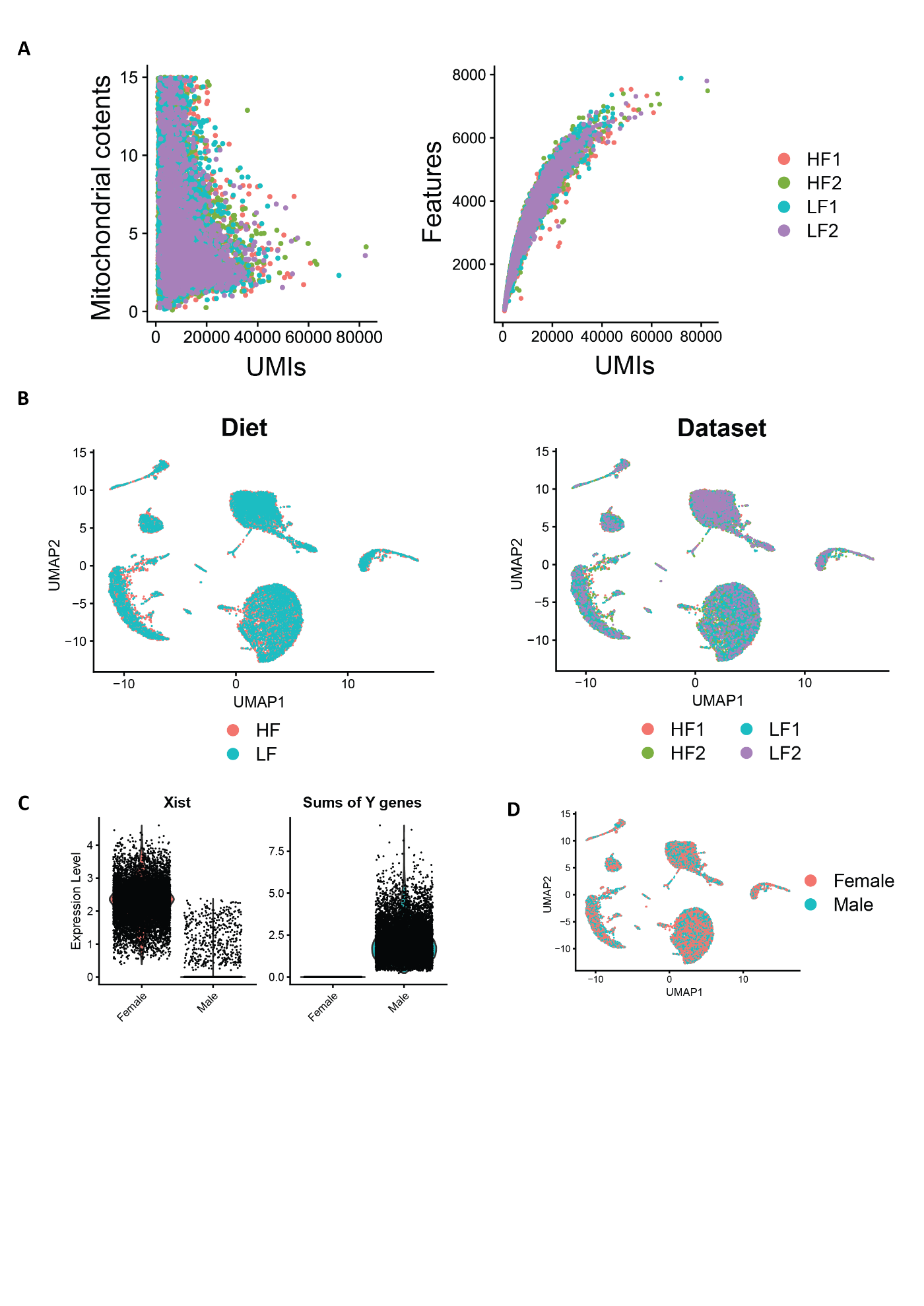


**Figure S3 | Quality control and sex identification of single-cell RNA (scRNA)-seq datasets. A.** Plot showing unique molecular identifiers (UMIs), gene counts, and mitochondrial RNA contents of cells. These data demonstrate high quality of scRNA-seq datasets. **B.** Uniform Manifold Approximation and Projection (UMAP) plots showing the distribution of cells from 4 datasets of 2 types of maternal diet. HF, high-fibre. LF, low-fibre. **C.** Expression of Xist and genes on Y chromosomes in droplets (cells) identified as male and female, respectively. **D.** UMAP plots showing the distribution of cells identified as male and female.


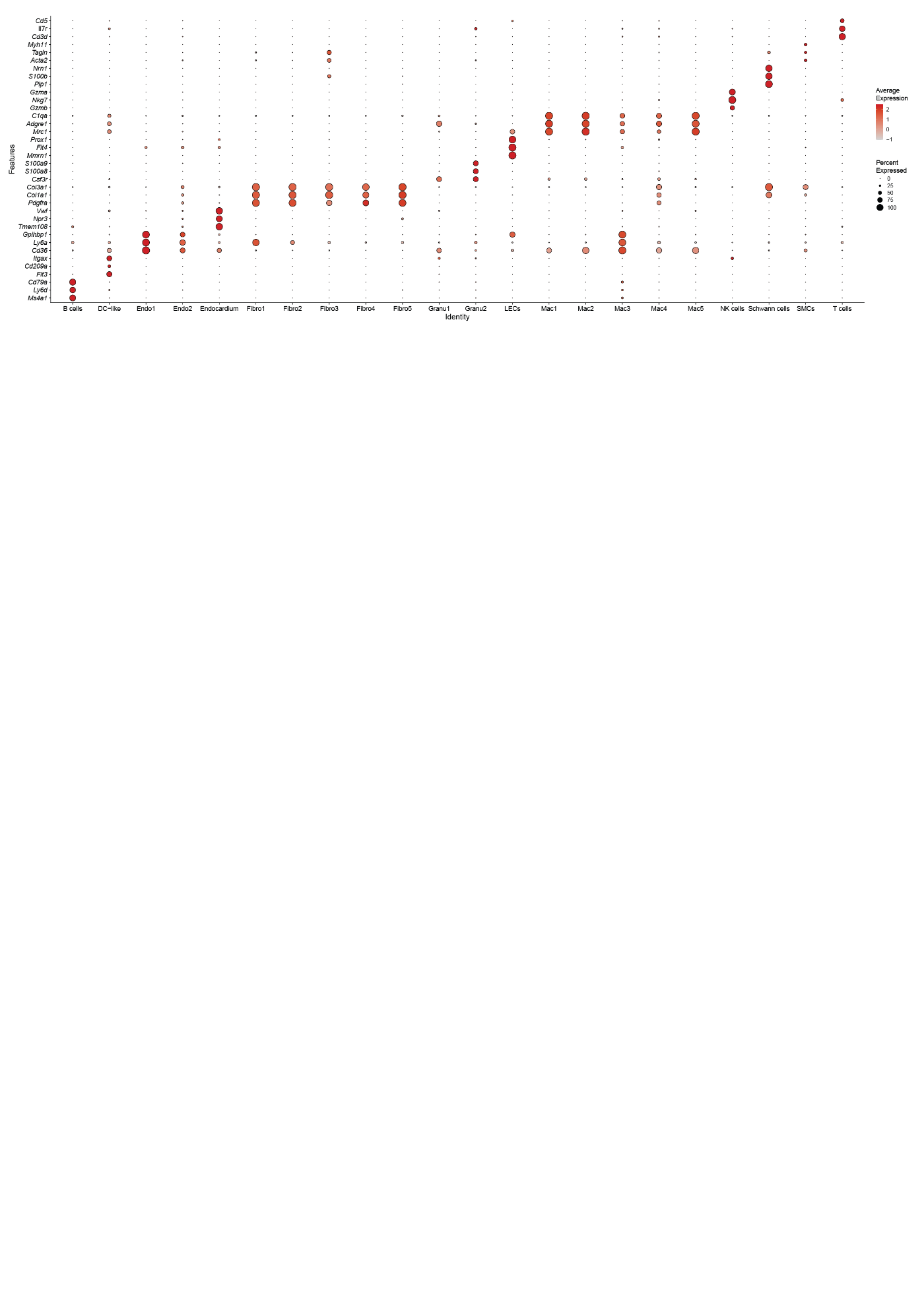


**Figure S4 | Dot plot showing marker genes for cell type annotation in the whole dataset.**


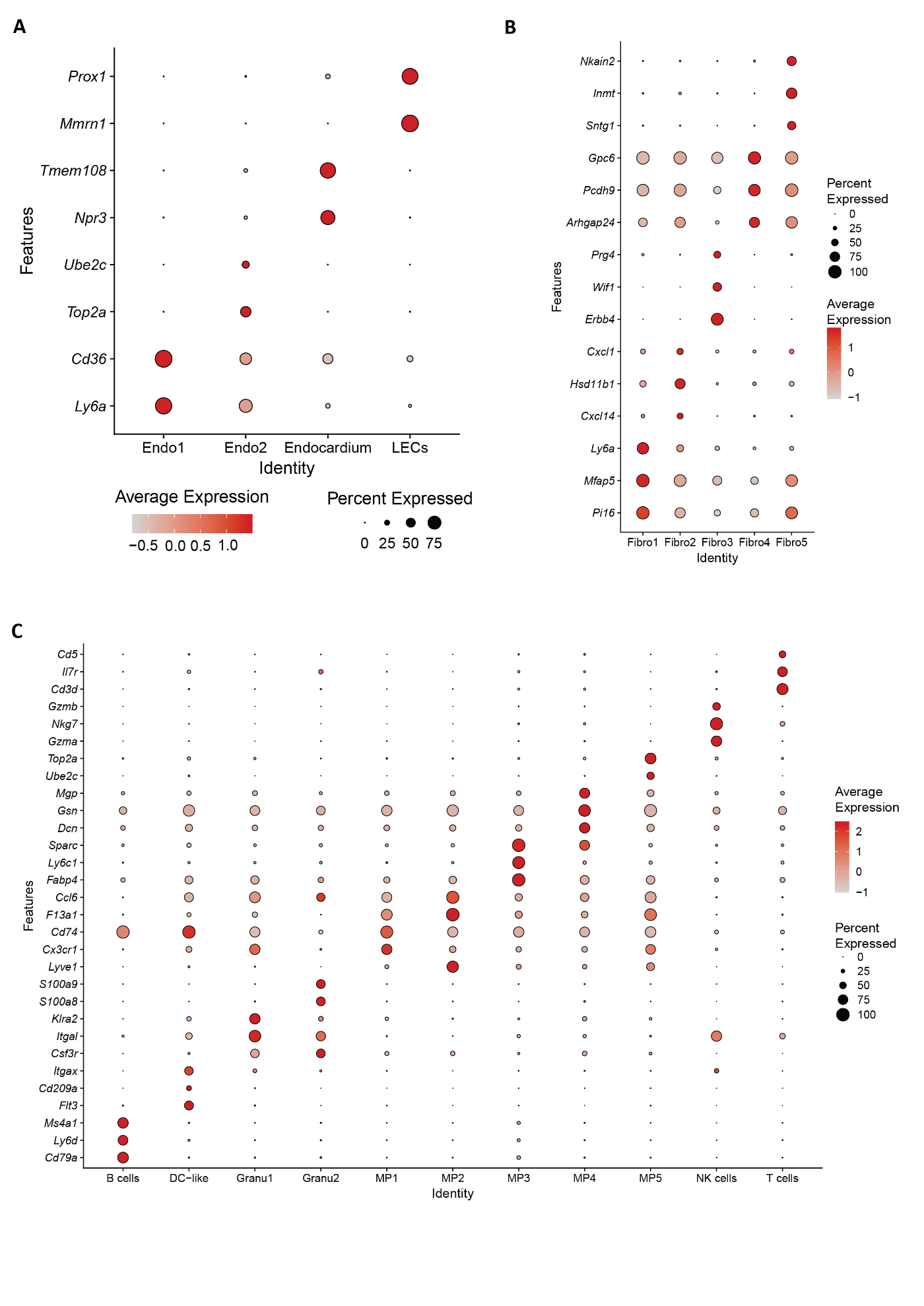
**Figure S5 | Marker genes for cell type annotation in: A.** endothelial marker-expressing clusters. **B.** fibroblast marker-expressing cluster. **C.** immune marker-expressing clusters.


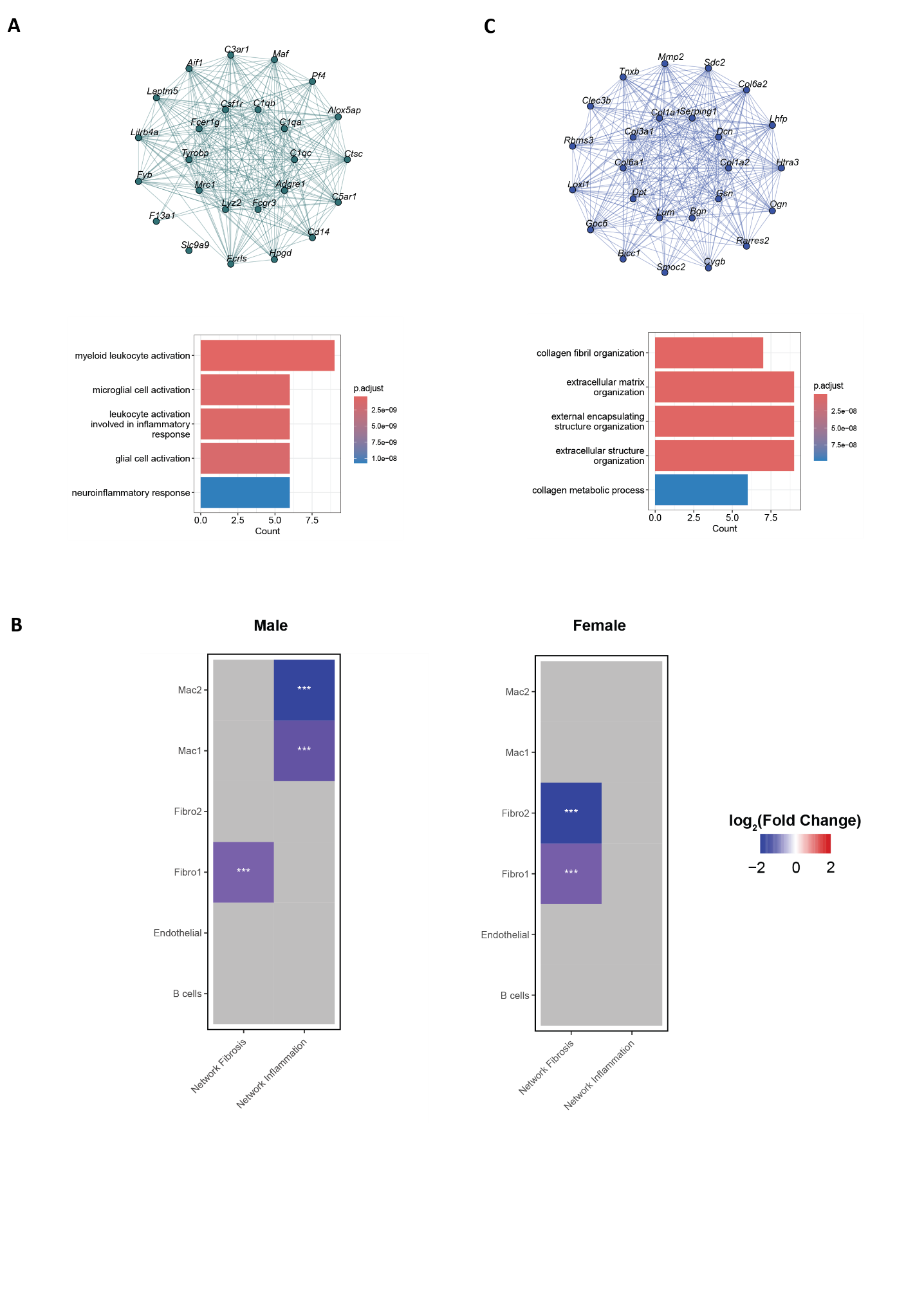


**Figure S6 | Changes in co-expression network of male and female of high-fibre offspring. A.** A co-expression network related to inflammation. **B**. heatmaps showing changes of expression levels of co-these two expression networks in male and female.**C.** A co-expression network related to fibrosis.


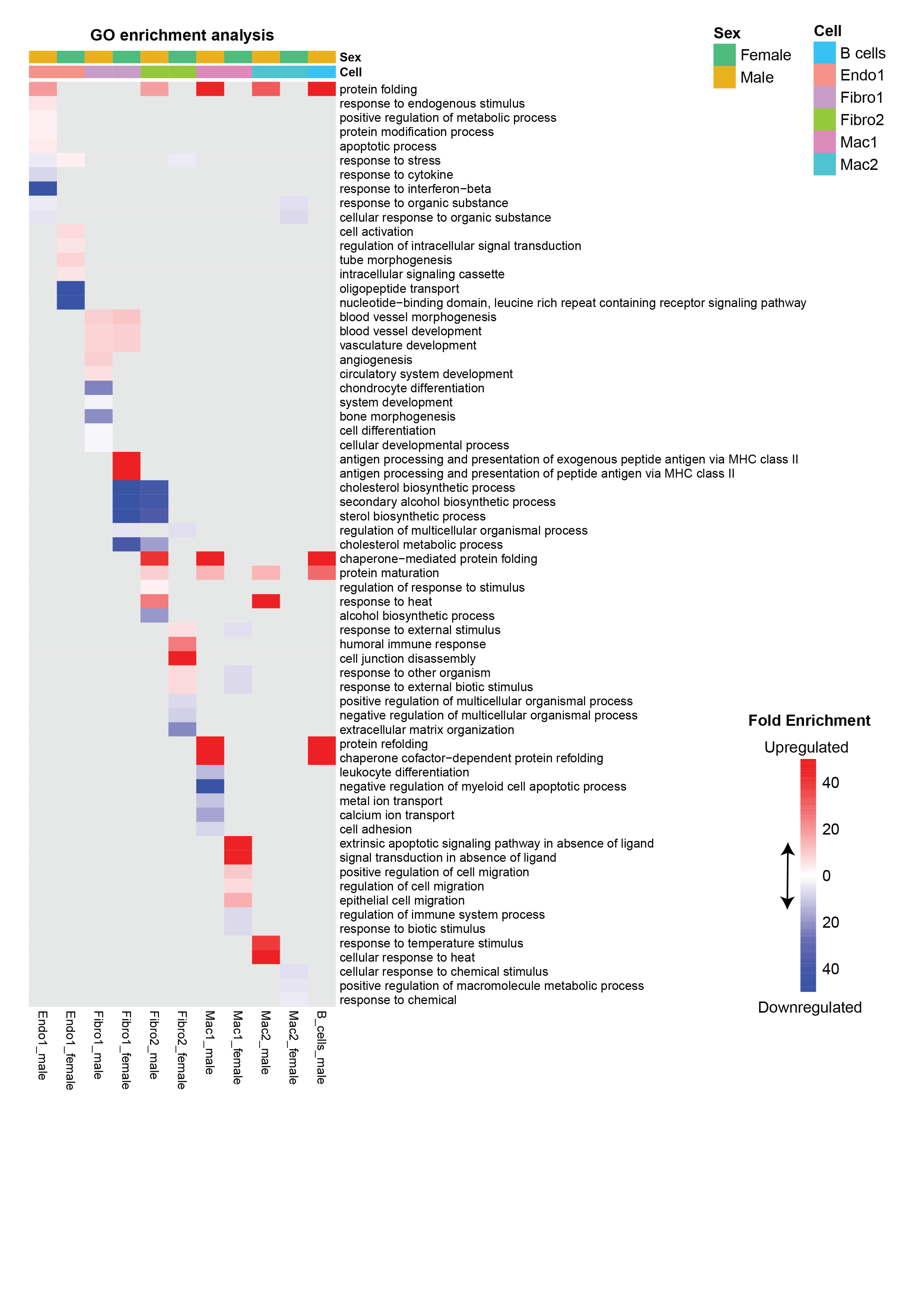


**Figure S7 | Comparison of the top 5 significantly enriched gene ontology (GO) Biological Process terms across different cell types in male and female offspring. If fewer than five significant terms were found, all are shown. Conditions with no significant enrichment or fewer than five differentially expressed genes (DEGs) were excluded from the plot. Fold enrichment of upregulated pathways was shown in red, and downregulated pathways in blue.**


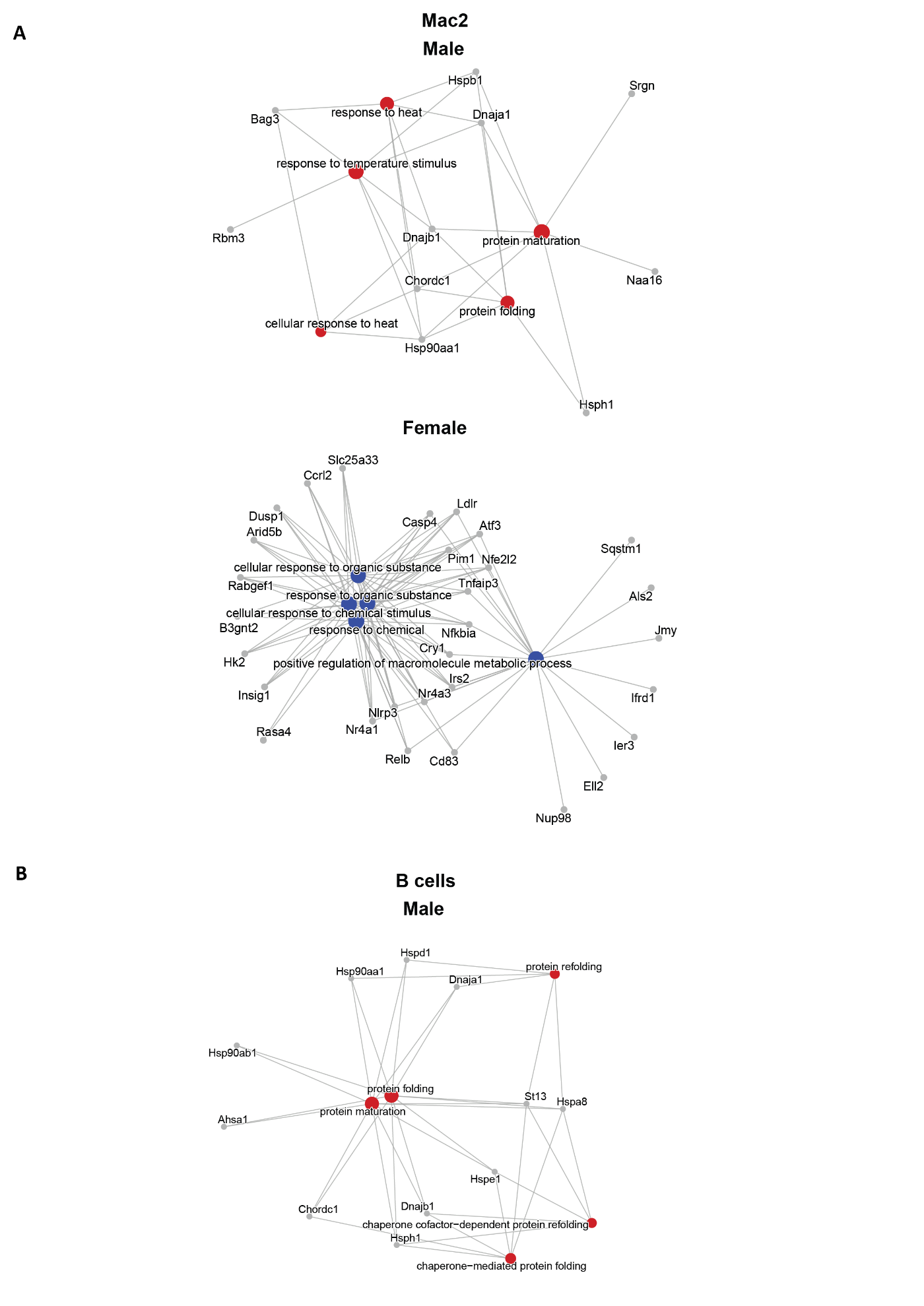


**Figure S8 | Pathway enrichment analysis for differentially expressed genes in A. Mac2 subpopulation** in male (only upregulated genes had significantly enriched result) and female (only downregulated genes had significantly enriched result)**. B. Upregulated genes in B cells of male HF offspring**. Analysis was not performed for downregulated genes in male HF offspring or any differentially expressed genes (DEGs) in female HF offspring due to a low number of DEGs (<5)**.**


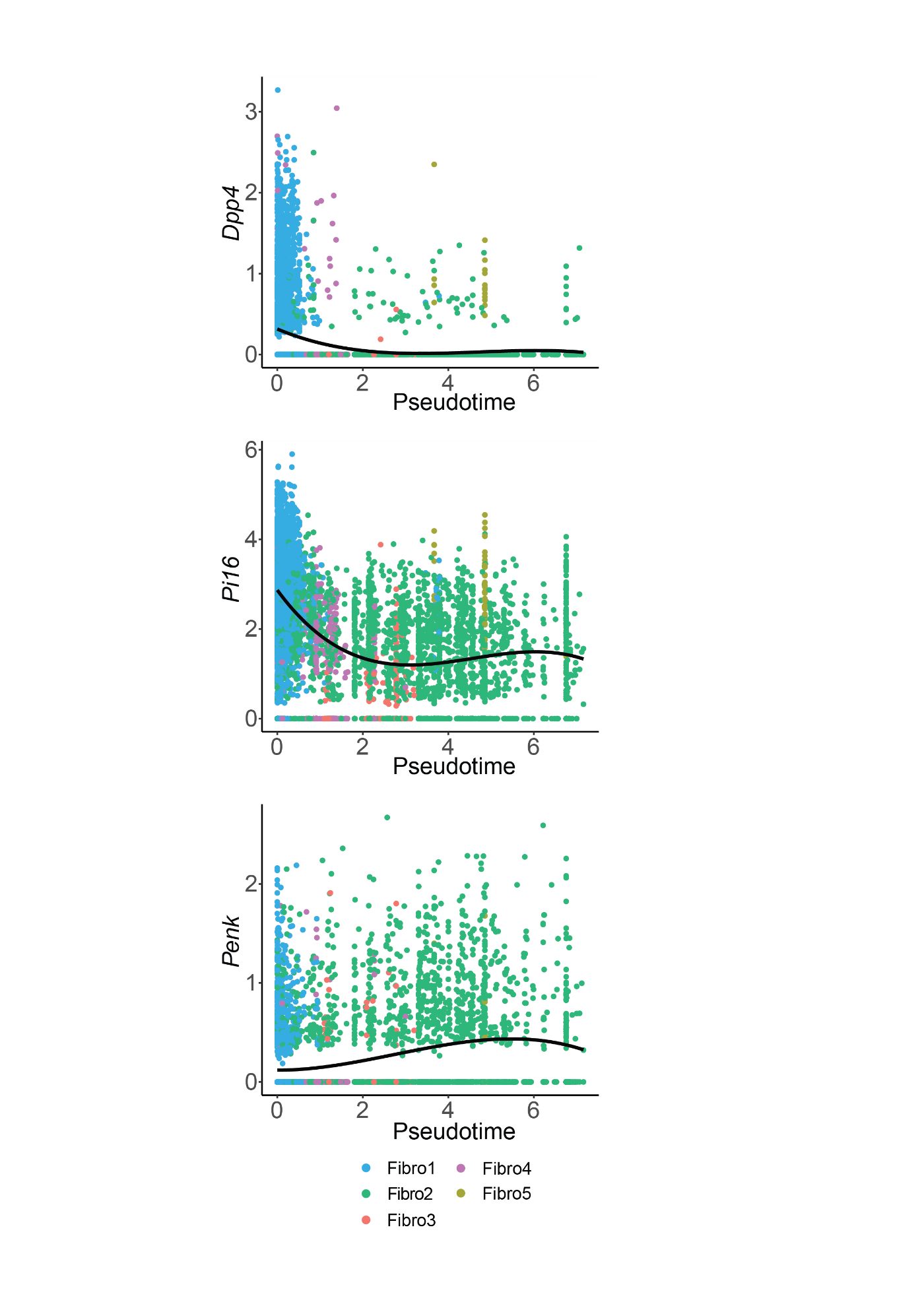


**Figure S9 | The expression of *Dpp4, Pi16,* and *Penk* genes along the reconstructed differentiation trajectory,** showing the expression of progenitor marker genes *Dpp4* and *Pi16 is* decreasing and the fibroblast marker *Penk* is increasing along the reconstructed differentiation trajectory*.*

**
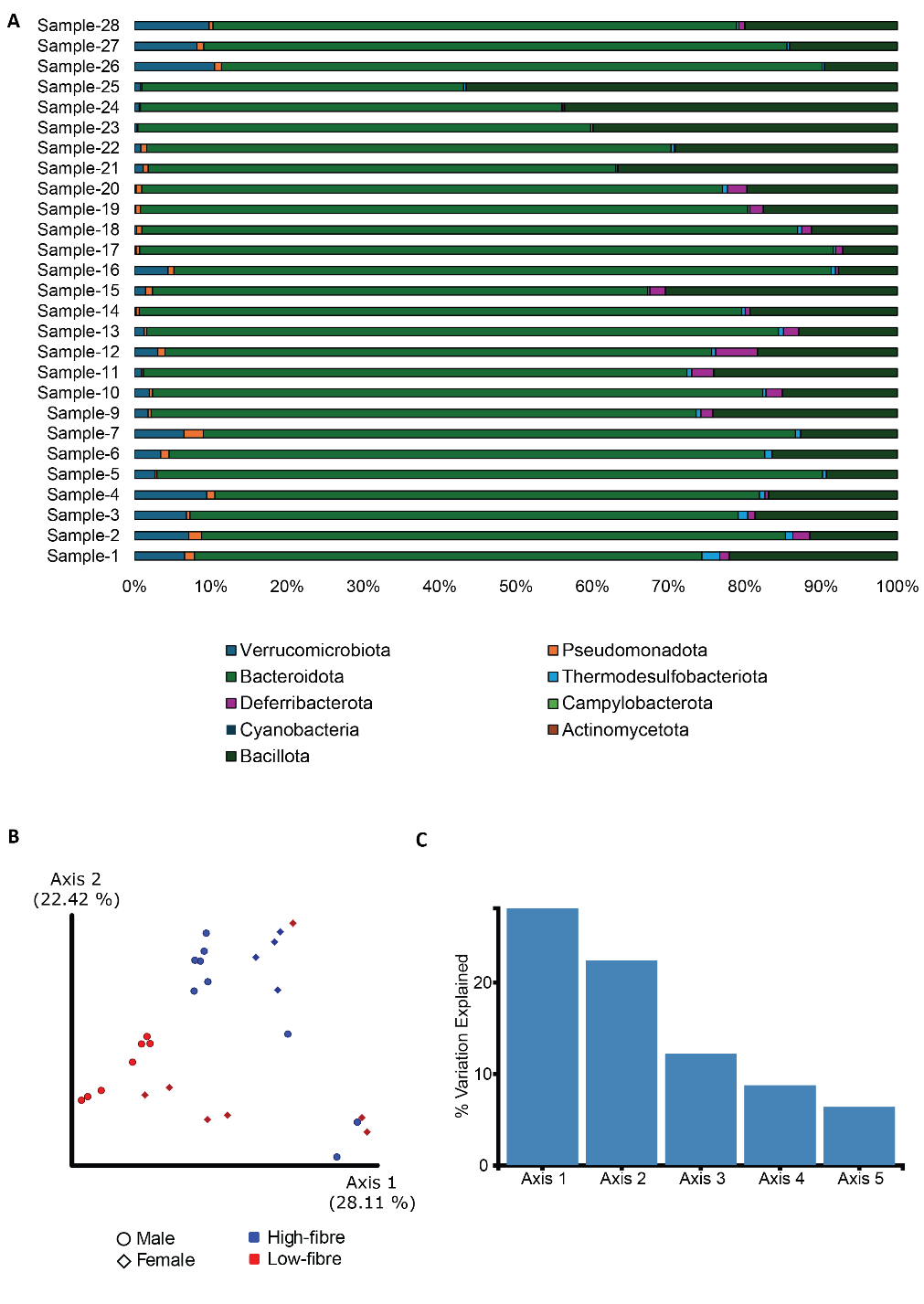
**

**Figure S10 | Taxonomy and diversity of the gut microbiome in male and female offspring. A.** Phylum-level taxa of 16S rRNA-seq samples. **B**. Principal coordinate analysis plot showing Bray-Curtis β-diversity of gut microbiome in male and female high-fibre offspring. **C.** Variance explained by axes of Bray-Curtis β-diversity PCoA analysis. Sample size: n=4 female high-fibre offspring, n=9 male high-fibre offspring, n=7 female low-fibre offspring, n=7 male low-fibre offspring.
